# Biomedical surface analysis: Evolution and future directions (Review)

**DOI:** 10.1116/1.4982169

**Published:** 2017-04-24

**Authors:** David G. Castner

**Affiliations:** National ESCA and Surface Analysis Center for Biomedical Problems, Molecular Engineering and Sciences Institute, Departments of Bioengineering and Chemical Engineering, University of Washington, Box 351653, Seattle, Washington 98195-1653

## Abstract

This review describes some of the major advances made in biomedical surface analysis over the past 30–40 years. Starting from a single technique analysis of homogeneous surfaces, it has been developed into a complementary, multitechnique approach for obtaining detailed, comprehensive information about a wide range of surfaces and interfaces of interest to the biomedical community. Significant advances have been made in each surface analysis technique, as well as how the techniques are combined to provide detailed information about biological surfaces and interfaces. The driving force for these advances has been that the surface of a biomaterial is the interface between the biological environment and the biomaterial, and so, the state-of-the-art in instrumentation, experimental protocols, and data analysis methods need to be developed so that the detailed surface structure and composition of biomedical devices can be determined and related to their biological performance. Examples of these advances, as well as areas for future developments, are described for immobilized proteins, complex biomedical surfaces, nanoparticles, and 2D/3D imaging of biological materials.

## I. INTRODUCTION

The beginning of modern biomedical surface analysis can be traced back several decades.^1–3^ While the origins and importance of surfaces have a much longer history, the earliest surface analysis studies on biomedical materials were done 30–40 years ago.^1–3^ These early biomedical surface analysis studies typically used a single technique, such as x-ray photoelectron spectroscopy (XPS, also known as electron spectroscopy for chemical analysis or ESCA), to investigate a homogeneous material.^4^ As polymers were used in some of the very first biomaterials and continue to be extensively used in biomedical applications, many of the early biomedical surface analysis studies were done on polymeric materials.^5–8^ These studies focused on characterizing polymers with well-defined functionalities (acrylics, fluorocarbons, aromatics, etc.) where the structure and functionality could be systematically varied. For example, the side chain of methacrylates can be varied in length (e.g., C_1_ to C_12_ alkyl chains) or character (e.g., alkyl to aromatic). Thus, the structure and composition of a given polymer system could be varied and the effect of that change is monitored using surface analysis (e.g., surface composition using XPS).

From these beginnings, biomedical surface analysis has expanded and increased in complexity in terms of both the techniques used, types of analyses carried out, and materials investigated.^9–11^ Throughout this evolution and development, the following general goals have provided guidance: (1) the surface region of a biomaterial is the interface between the biomaterial and the biological environment, mediating the biological response (protein adsorption, cell attachment, etc.) to the biomaterial; (2) the composition, structure, orientation, and spatial distribution of surface species play an important role in biological reactions with biomaterials; and (3) state-of-the-art instrumentation, experimental protocols, and data analysis methods are required to provide detailed analysis of biomaterial surfaces and interfaces.

The National ESCA and Surface Analysis Center for Biomedical Problems (NESAC/Bio) provides an example of how biomedical surface analysis evolved in terms of expanding from single technique analysis to complementary, multitechnique analysis and from homogeneous, simple materials to complex, biological materials. NESAC/Bio was founded by Professor Buddy Ratner in 1983 with funding from the U.S. National Institutes of Health. It started as a center with one XPS instrument and a focus on polymer surface analysis. Over the past 30+ years, a wide range of analysis techniques such as secondary ion mass spectrometry (SIMS),^12,13^ atomic force microscopy (AFM),^14^ near-edge x-ray adsorption fine structure (NEXAFS),^15^ surface plasmon resonance (SPR),^16^ sum frequency generation vibrational spectroscopy (SFG),^17^ and quartz crystal microbalance with dissipation (QCM-D)^18^ have been added and used to provide comprehensive, complementary analysis of surfaces and interfaces. Each of these techniques has their own strengths and limitations as well as different sampling depths, but together they can provide a comprehensive analysis of biomedical surfaces. Along with the expanded number of techniques, significant advances in the capabilities of a given technique have also been realized. For example, SIMS analysis started with a quadrupole mass analyzer and an atomic noble gas primary ion beam mounted onto the XPS instrument and then evolved using various stand-alone instruments with capabilities that now include multiple primary ion sources (liquid metal, gas cluster, and C_60_ ion beams), time-of-flight (ToF) mass analyzers, ms/ms detection, and sophisticated sample handling. In addition, experimental protocols such as frozen-hydrated^19^ and trehalose coating^20^ were developed to prepare biological samples for analysis in ultrahigh vacuum (UHV) conditions. Methods were also developed for analyzing the wealth of data produced by all these biomedical surface analysis techniques. Examples include generating depth profiles from angle-dependent XPS data^21^ and multivariate analysis (MVA) processing of ToF-SIMS data.^22^ These advances have allowed the complexity of the samples analyzed to continually expand from polymers to RF glow discharge deposited films to self-assembled monolayers (SAMs) to biorecognition materials to DNA/protein microarrays to biological cells/tissue sections to nanoparticles (NPs). Concurrent with the expansion of sample complexity has been the evolution from spectroscopic analysis of homogenous surfaces to imaging of patterned 2D surfaces to depth profiling and 3D imaging of organic and biological materials.

**Fig. 1.**
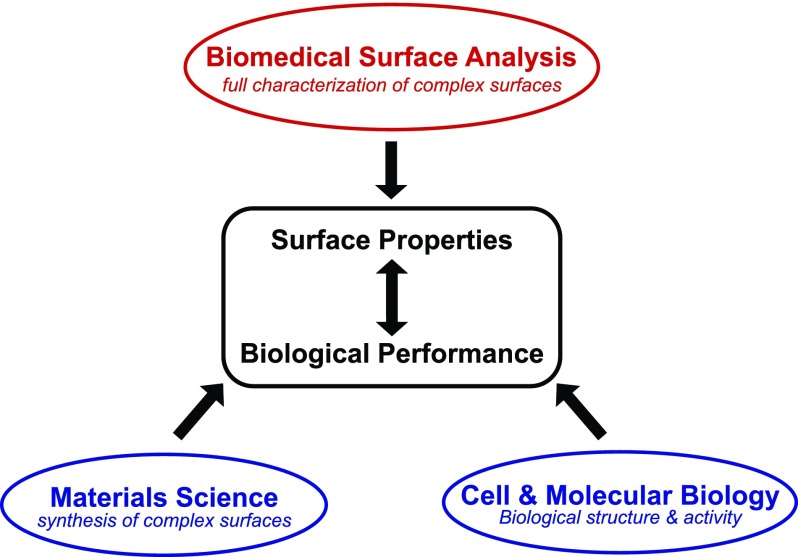
Diagram showing the integration of biomedical surface analysis with materials synthesis and biological studies to investigate the relationship between surface properties and biological performance.

Although the subject of this review is focused on developing biomedical surface analysis tools for detailed, multitechnique characterization of complex, biological materials, it is important to remember that to maximize the impact of surface analysis of biomedical devices, it needs to be integrated with materials synthesis and biological studies, as depicted in Fig. 1. Bringing all the aspects of biomedical devices together will provide us a better understanding of how the surface properties of a biomaterial affect its biological performance, thereby allowing one to design biomaterials with improved and novel biological properties. With that in mind, the rest of this review will focus on four areas of biomedical surface analysis to provide examples of the advances that have been achieved over the years as well as the opportunities that exist for future advances. While these examples will largely be drawn from the research done at NESAC/Bio for the past 30+ years, there are similar examples and advances that have also been made by other biomedical surface analysis research groups around the world during this time period.

## II. CASE STUDIES IN BIOMEDICAL SURFACE ANALYSIS

Biomedical surface analysis can be applied to a wide range of problems and materials. Some selected ultimate goals for biomedical surface analysis include: (1) atomic level structure determination of surface-bound biomolecules (proteins, peptides, DNA, lipids, etc.), (2) detailed characterization of complex, multicomponent systems, (3) *in situ* characterization of NPs, and (4) imaging of biological cells and tissue sections. Various degrees of progress have been made toward of these goals. In Secs. II A–II D, examples, accomplishments, and challenges for each of these goals will be discussed.

### A. Characterization of surface bound proteins

**Fig. 2.**
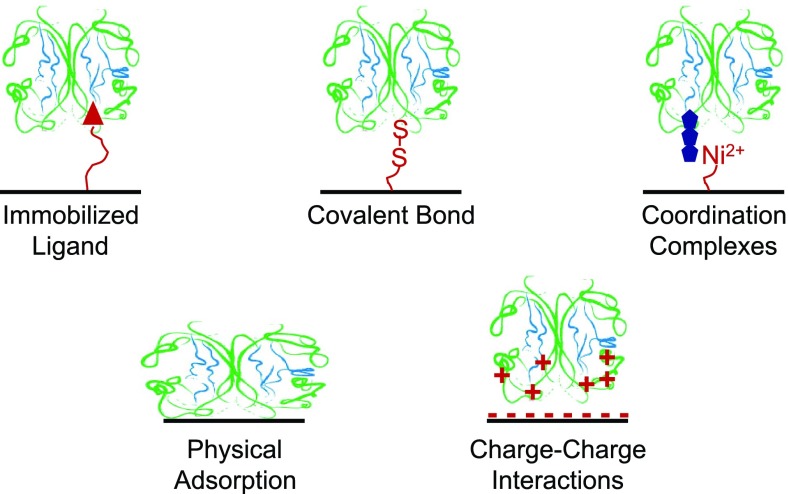
Some protein immobilization schemes commonly used to attach proteins to surfaces.

One of the first events that occurs once a biomedical device is placed in the biological environment is the interactions of proteins with the surface region of the biomedical device.^23^ How the proteins interact with the surface can have a significant impact on further biological responses such as cell attachment, biofilm formation, and biomineralization in both *in vivo* and *in vitro* applications.^24^ Protein surface interactions also play an important role in diagnostic assays such as enzyme-linked immunosorbent assay.^25^ Thus, it is essential to understand how proteins interact with surfaces and any structural modifications they undergo as a result of these interactions. Key objectives for characterizing surface-bound proteins are (1) identifying the type of protein bound to the surface, (2) determining the amount of each surface-bound protein, (3) determining the conformation and orientation of the bound proteins, and (4) characterizing the spatial distributions of surface-bound proteins.^26^ There are many bonding mechanisms for attaching proteins to surfaces,^27^ including charge-charge, coordination complexes, covalent bond formation, and ligand interaction schemes, as shown in Fig. 2. Each method has its advantages and disadvantages. How the protein structure, especially its conformation and orientation, is affected by surface attachment will be a function of the surface structure and composition of the biomaterial (e.g., type and distribution of functional group in the surface region) as well as the properties of the protein (rigidity, charge distribution, etc.). There are often time-dependent changes in the composition, conformation, orientation, and distribution of the complex, multicomponent protein films deposited from the biological environment.^28,29^ So, the structural determinations for surface bound proteins need to be related not only to the properties of the biomaterial surface and protein but also to the experimental conditions (time, solution concentration, solution *p*H, etc.) used to attach the protein to the surface.

The seminal publications of Ratner and colleagues represent some of the first surface analysis studies of protein films.^30,31^ Their studies in the early 1980s using angle-dependent XPS and radiolabeling to characterize protein films on polymer surfaces laid out the principles for analyzing surface bound proteins using XPS. Recent studies have been revisited using XPS to measure the thickness of adsorbed proteins in combination with other techniques such as spectroscopic ellipsometry and QCM-D.^32^ The combination of ^125^I radiolabeling with XPS represents a powerful method for determining the thickness of adsorbed protein films. ^125^I radiolabeling of proteins is the “gold standard” for measuring the amount of protein adsorbed onto a surface.^33^ The absolute amount of adsorbed protein from ^125^I experiments is then used to calibrate the relative surface composition determined by XPS (atomic % N, C, O, etc.) and calculate the thickness of the protein film.^34^ These calculations relied on having an element that was only present in the protein overlayer (e.g., N) and not in the polymer substrate. More complex systems where there is no element that is unique to the protein overlayer (e.g., N present in both the protein film and the substrate) require additional calculations to account for the N substrate contribution to the XPS signal.^35^ The approach of using radiolabeling experiments to calibrate the XPS composition has also been extended to other biomolecules such as DNA.^36,37^

**Fig. 3.**
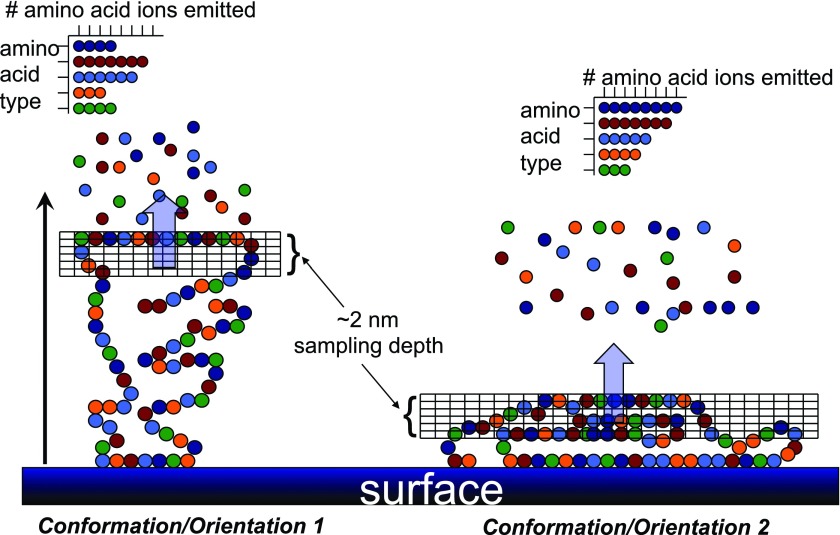
Schematic diagram showing how a surface-bound protein with a nonuniform 3D amino acid distribution in two different conformations/orientations can produce different distributions of the amino acid fragments detected in a static ToF-SIMS experiment.

Although XPS can provide important information about protein films such as thickness, the fact that all proteins have similar elemental concentrations of carbon, oxygen, and nitrogen limits the usefulness of XPS for distinguishing between different proteins, let alone investigating the conformation and orientation of proteins. For example, the XPS C_1s_ spectra of pure protein films can exhibit small differences in the peak areas of the individual carbon species (C–C/C–H, C–O/C–N, and O=C–N/O=C–O),^38^ but these small differences are insufficient to distinguish samples that contain more than one protein or differentiate them from substrates that contain similar organic functional groups. Thus, additional surface analysis methods that have greater sensitivity to protein structures needed to be developed. Another seminal publication by Ratner and colleagues unlocked the possibilities of using SIMS to characterize surface-bound proteins.^39^ The key finding of this study was that each amino acid residue in a protein produced unique secondary ion fragments. This implies that from a SIMS standpoint, proteins could be viewed as polymers with 20 different monomer units and all the analysis methodology developed for polymer analysis using SIMS could also be applied to protein analysis. Most importantly, although most proteins contain the same 20 amino acids, the relative concentration of each amino acid as well as their locations in the protein 3D structure varies from protein to protein. Since static SIMS directly probes the surface amino acid concentration of a protein, this allows static SIMS to be used to determine the identity, concentration, conformation, orientation, and spatial distributions of surface-bound proteins.^26^ Another key point is that the molecular fragment sampling depth of SIMS in the static mode is ∼2 nm,^40^ which is smaller than characteristic dimensions of most proteins. So, the amino acid fragment intensity pattern is not only sensitive to the type of protein present on the surface but also sensitive to its conformation and orientation.^38^ A schematic depicting the surface sensitivity of static SIMS and how it affects the amino acid fragment intensities is shown in Fig. 3. It is important to remember that the largest difference in the amino acid fragment intensity patterns is from one protein to another protein.^28^ So, although changes in protein conformation and orientation are detectable using SIMS, the differences in the amino acid fragment pattern from different protein conformations and orientations should be considered a second order effect relative to differences in amino acid concentrations among different types of proteins.

Most amino acids can produce two or more unique SIMS fragments.^39^ So, to fully exploit the amino acid fragment intensity pattern differences of proteins, the intensity variations of ∼40 fragments need to be monitored and analyzed. MVA, particularly principal component analysis (PCA), is an approach that has been typically used to address this challenge.^22^ In addition, to overcome the biological variation that is typically present in protein adsorption experiments, it is essential to acquire a significant number of replicates. For ToF-SIMS analysis of protein films, this means analyzing 3–5 replicates per sample type and 3–5 spots on each replicate to provide at least a minimum of nine datasets per sample type. By using ToF-SIMS with PCA to process the amino acid fragment intensities, it was shown that different adsorbed protein films could be readily distinguished based on differences in the amino acid concentrations of different proteins.^28,41^ This not only was possible for different types of proteins (e.g., albumin versus fibrinogen) but also allowed the same type of protein from different species to be differentiated (e.g., albumin from bovine, chicken, porcine, and human).^28^ Once it was established that the combination of ToF-SIMS with MVA allowed different proteins to be identified, then the next step was to quantify the amount of each protein present in a mixed protein film. A multitechnique approach of ToF-SIMS with partial least square regression, XPS, and ^125^I radiolabeling was used to investigate binary and ternary mixed protein films.^34^ The results showed that as long as a protein was present at concentrations of at least 10 mass % in the mixed protein film, it could be identified and quantified. This implies that if a mixed protein film has ten different proteins with each protein present at a 10 mass % level, then each protein could be identified and quantified. However, it would be challenging to fabricate a mixed protein film with exactly 10 mass % of each protein. So, a more realistic limit of the complexity of mixed protein films that should be quantitatively analyzed using ToF-SIMS and MVA is probably four or five different proteins. However, even though quantitative analysis of protein films deposited from complex mixtures such as blood plasma or serum is not possible, qualitative trends can be observed in these complex protein films.^29^

Using ToF-SIMS with MVA to determine the identity and concentration of a protein only relies on the differences in the bulk amino acid concentrations between proteins. Thus, for these investigations, it is not necessary to maintain the 3D structure of the surface-bound proteins when the sample is dried and transferred into the UHV environment for ToF-SIMS analysis. However, if the conformation and orientation of a surface-bound protein are to be determined, then the 3D structure of the surface-bound protein must be maintained while the sample is dried and analyzed in UHV. Some methods that have been developed to preserve protein structures under these conditions include trehalose coating of the proteins,^20,42^ cross-linking of the proteins,^43,44^ and using poly(ethylene glycol) (PEG) based surfaces.^35^ The additional requirements for these investigations are that the protein being investigated has a heterogeneous distribution of one or more amino acids across its 3D structure and that the 3D structure is known. For example, in many blood proteins, the hydrophilic amino acids are preferentially located on the outer surface of the protein while the hydrophobic amino acids are preferentially located in the inner core of the protein. For these proteins, it is often possible to track the intensity ratio of hydrophilic to hydrophobic amino acid fragments to follow protein denaturation processes.^20^ There can be exceptions to these general trends. For example, the hydrophobic amino acid valine is preferentially located near the outer surface of albumin.^35^ So, for a complete interpretation of the ToF-SIMS results, it is always best to start with the 3D protein structure from the Protein Data Bank (http://www.rcsb.org/pdb/home/home.do) and determine the relative amino acid distribution across the 3D structure. Orientation information can be obtained in a similar manner by following the intensities of fragments from amino acids that are preferentially located on one side or region of the protein.^45^

The first studies using ToF-SIMS to examine protein conformation and orientation were on proteins that had sizes that were large (>5 nm) relative to the static ToF-SIMS sampling depth of ∼2 nm.^20,43,46,47^ More recent studies investigated using ToF-SIMS the orientation of smaller proteins and peptides. The orientation of Protein G, a barrel shaped protein with a length of ∼3 nm that is similar to the static ToF-SIMS sampling depth, was successfully determined using intensity ratios from amino acid preferentially located in opposite ends of the protein.^9,48^ When 25 keV Bi^+^ or Bi_3_^+^ primary ion beams are used for these experiments, the difference in intensity ratios is small (15%–20%) but significant. Much larger differences (∼75%) are observed if 20 keV Ar_1000_^+^ gas cluster primary ion beams are used to examine Protein G orientation. Also, isotope labeling of key amino acid fragments (e.g., ^13^C labeled methionine) can be used to produce increased sensitivity to Protein G orientation.^49^ The orientation of peptides with even smaller dimensions has also been examined. Leucine (L) and lysine (K) containing peptides that form α-helix and β-sheet structures with L side chains on one side of the peptide backbone and K side chains on the other side are good model systems for these studies.^50^ For the β-sheet LK peptide, the ToF-SIMS K/L intensity ratio exhibited large and significant differences when the peptide was adsorbed onto methyl versus carboxylic acid SAMs. Thus, in spite of the fact that the LK peptide thickness is <2 nm, ToF-SIMS can be used to determine its orientation when it forms a β-sheet structure and the L and K side chains are pointed ∼180° from each other. For the LK peptide with the α-helix structure, the peptide side chains fan out forming polar and apolar hemicylinders. ToF-SIMS does detect small differences in the K/L intensity ratio for the α-helix LK peptide adsorbed onto methyl versus carboxylic acid terminated SAMs, but the difference is similar to the standard deviations of the ToF-SIMS measurements. Thus, when the biomolecule dimensions are at or below the ToF-SIMS sampling depth, the ability of ToF-SIMS to determine the orientation will depend on how well separated the characteristic amino acids are in the structure. Gas cluster ion beams offer the potential to more definitively examine the orientation and conformation of biomolecules with smaller size dimensions.

**Fig. 4.**
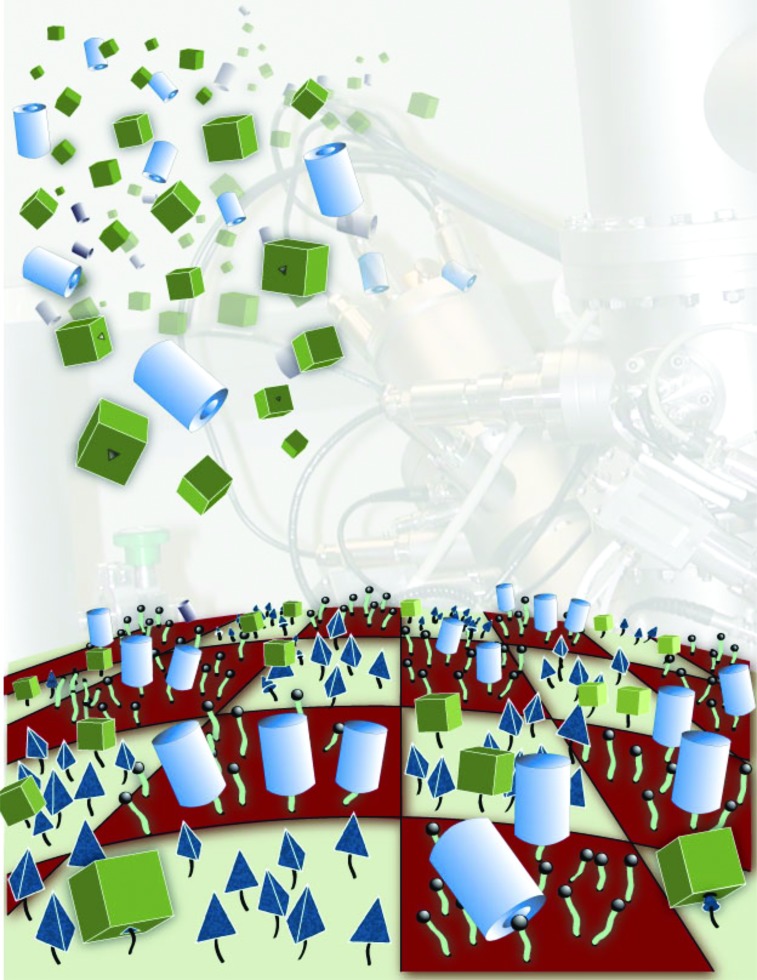
Schematic diagram of a surface with two different ligands patterned onto the surface of a PEG-based polymer then exposed to mixed solution of two proteins that are selectively bound to the patterned ligands and imaged using ToF-SIMS.

The examples in the previous paragraphs discussed the advances made in characterizing the identity, concentration, conformation, and orientation of surface-bound proteins, which was done using homogeneous, uniform surfaces. The next level of complexity is to obtain the same level of information about the structure of surface-bound proteins but in a spatially resolved manner. One route for accomplishing this is to prepare a patterned surface with different protein binding species present in different regions, as depicted in Fig. 4. To minimize nonspecific protein adsorption, the binding species should be attached to a protein resistant background (e.g., a PEG surface). In one example, biotin and chloroalkane ligands were patterned onto the surface of a PEG-based polymer using standard photolithography methods, and then, the surfaces were exposed to a mixed solution of proteins that specifically bind to each ligand (streptavidin to biotin and HaloTag to chloroalkane).^51^ Using the differences in amino acid concentrations between streptavidin and HaloTag, ToF-SIMS imaging showed the proteins selectively bound to their respective ligands. In a similar experiment, Protein A and fluorescein were patterned onto a PEG-based polymer to control the orientation of an immunoglobulin G (IgG) antibody.^52^ Using the differences in the amino acid composition of the F_ab_ and F_c_ regions of IgG, ToF-SIMS demonstrated that IgG was preferentially bound with the F_ab_ region facing away from the surface in the Protein A regions and facing toward the surface in fluorescein regions.

Significant progress has been made in developing surface analysis methods such as ToF-SIMS for characterizing the structure of proteins immobilized onto flat surfaces. Future biomolecule characterization needs include extending these studies to more complex samples (NPs, porous tissue scaffolds, drug loaded stents, etc.) as well as more tightly integrating complementary techniques such as SFG that can be used to study immobilized biomolecules in the presence of the biological environment. In addition, further advances in computational methods for predicting biomolecule–surface interactions and structures as well as providing structural information at the atomic level for large biomolecules are needed.^53^

### B. Characterization of complex surfaces

The ultimate goal of characterizing complex surfaces is not only to provide a detailed identification of all the different species present near the surface of a multicomponent sample but also to perform this in a spatially resolved manner so the concentration of each species can be specified at least in two dimensions across the surface and often using depth profiling to add the third dimension to the sample. For biomaterials fabricated from some combination of multicomponent polymers, metals and ceramics, this typically means identifying all the chemical species present and their 2D or 3D distributions. For devices containing biomolecules (drug eluting stents, surface immobilized proteins, etc.), this typically means also characterizing the biomolecules present in terms of identity and structure (conformation, orientation, etc.), as described in Sec. II A.

In addition to looking for the chemical species and biomolecules expected to be present based on the design of the biomedical device, it is also essential to look for unexpected species introduced by the fabrication process or exposure to various environments.^54–57^ For example, release agents and lubrication films can be transferred from processing equipment to the sample, airborne adventitious hydrocarbon and poly(dimethyl silicone) (PDMS) species can be deposited onto the sample, and chemical reactions such as surface oxidation of metals can occur on some samples. Often, these unexpected contaminants are easy to detect. For example, extremely low levels of PDMS can be easily detected using ToF-SIMS as all ToF-SIMS analysts are very familiar with the characteristic positive secondary ions from PDMS (m/z = 73, 147, etc.).^58^ In other cases, the species introduced by unexpected side reactions, deposition from processing solutions, etc. can have compositions and structures similar to the material being analyzed, making it challenging to identity these contaminants. For example, detection of a submonolayer amount of a photo-resist residue on the polymer substrate (i.e., an organic contaminant on an organic substrate) can be challenging.^54^ If the organic contaminants have a slightly different structure from the organic substrate, then ToF-SIMS is often the best technique to use. However, to ensure that all unexpected species are identified, it is often necessary to use MVA methods to process the entire set of ToF-SIMS peaks.

When characterizing complex, multicomponent samples, MVA methods can help identify differences among samples and some of the reasons for those differences.^29^ However, it is often the best approach to start with well-defined samples and then systematically increase the complexity of the samples. Developing the surface analysis methodologies and analysis procedures with well-defined samples is an excellent way to determine the extent of detailed information that can be obtained from each surface analysis technique for a particular type of sample. Then, as the sample complexity is increased by adding more species and varying their spatial distribution, the information developed with the well-defined samples can be used to obtain as much detail as possible about complex samples.

**Fig. 5.**
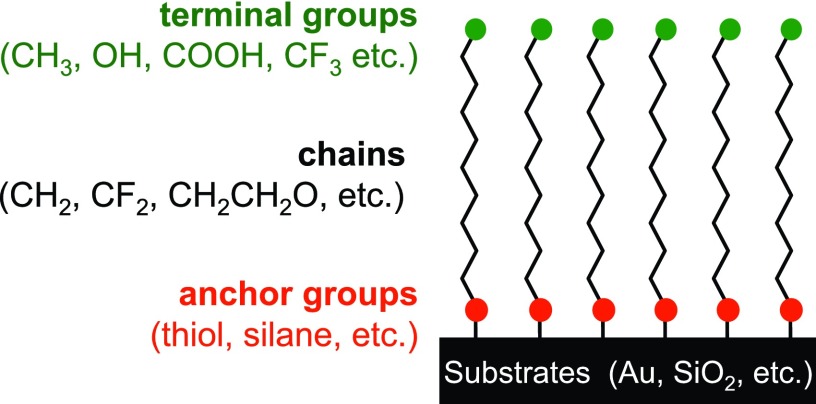
Schematic showing options for systematically varying the functionality and the structure of well-defined SAMs.

**Fig. 6.**
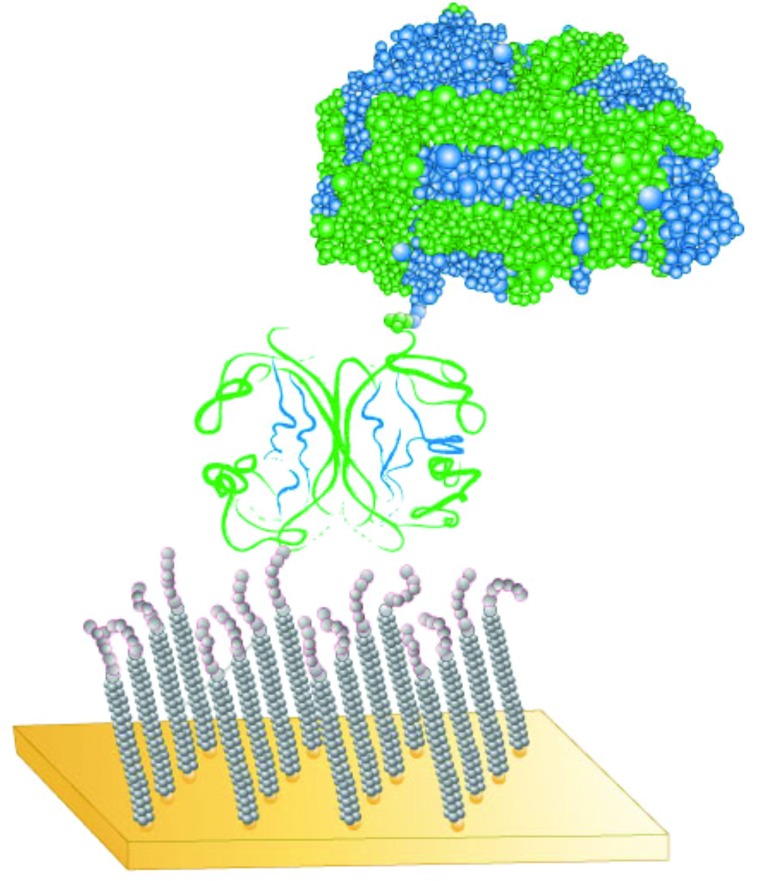
Schematic of the multicomponent sample prepared by first assembling a mixed SAM layer onto a gold surface, then attaching a streptavidin layer to the mixed SAM, and finally attaching a biotinylated protein to the streptavidin layer.

An excellent starting platform for well-defined samples is SAMs (see Fig. 5). A variety of anchor groups (thiols for gold, silanes for oxides, etc.) can be used to attach the molecules to the sample surface.^59–62^ The length and the type of chain (alkyl, ether, fluorocarbon, etc.) can be systematically varied. The terminal surface group (CH_3_, COOH, etc.) can also be systematically varied. The classic SAMs formed from assembling methyl terminated alkane thiols onto Au surfaces are a great platform for starting with a well-defined structure and then systematically increasing sample complexity.^63^ In these SAMs, the thiol binds to the Au surface, forming a Au-thiolate chemisorption bond.^64^ For chain lengths of at least 12 CH_2_ groups, the alkyl chains are well ordered and tilted 30°–35° from the surface normal.^63^ Thus, the structure and the thickness of the CH_3_-terminated, alkyl thiol SAMs are well defined. Techniques such as XPS, ToF-SIMS, and ellipsometry can be used to follow the changes in the thickness of the SAM overlayer as a function of the chain length.^65^ Methods such as SFG vibrational spectroscopy, Fourier transform infrared vibrational spectroscopy, and NEXAFS can be used to monitor the ordering and chain tilt angle in the SAM.^66^ The first step in increasing the complexity of these SAMs is to replace the terminal CH_3_ with another functional group. SAMs with reactive terminal groups can be used to investigate chemical reactions such as the derivatization of OH terminated SAMs (Ref. 67) and biomolecule immobilization onto SAMs.^68,69^ Another step in increasing the complexity is varying the type of group in the chain. Replacing the CH_2_ groups with CF_2_ groups changes the structure and the tilt angle, but they can still be well ordered.^70^ Using ether groups (CH_2_CH_2_O) in the chain typically introduces significant disorder into the SAM but can provide resistance to nonspecific protein adsorption.^71^ Other ways to introduce additional complexity into SAMs is to use mixed thiols (different chain length thiols, different terminal groups, different chain types, etc.) to produce a mixed SAM.^72,73^ Also, polymeric thin films, analogous to SAMs, can be used to add multiple components to the overlayer.^74,75^ For example, a fraction of the monomer units in a siloxane polymer backbone can be functionalized with alkyl disulfide side chains while another fraction of the monomer units is functionalized with PEG or fluorocarbon chains. This multicomponent polymer then assembles onto the surface in a three layered structure, with the disulfides forming Au-thiolate bonds to tether the polymer to the surface, then the siloxane backbone forming the middle layer, and finally the outer surface layer formed by the second side chain (PEG, fluorocarbon, etc.).^76^ While the polymeric thin films are not as well-ordered as traditional SAMs, they provide the increased versatility of polymers in terms of options for varying functionalities and structures. SAMs are a great starting point for preparing complex samples in a multistep process (see Fig. 6). For example, the first step could be assembling a mixed SAM with biotin and PEG terminal groups.^71^ In the second step, the protein streptavidin is attached to the biotin/PEG mixed SAM. Then, in the third step, a biotinylated protein is attached to the streptavidin layer. Each step in the process along with the final multicomponent sample can be characterized using a range of surface analysis techniques (XPS, ToF-SIMS, SPR, NEXAFS, etc.) to gain a detailed understanding of their composition and structure.^71,77^ Additional complexity can be introduced by going from flat, homogeneous surfaces to flat, patterned surfaces to highly curved, NP surfaces. Surface characterization of NPs will be discussed in Sec. II C.

Although significant advances have been achieved in obtaining detailed characterization of complex, multicomponent surfaces, there is always a need to continue to expand our capabilities for investigating these surfaces, especially carrying out these investigations in the presence of the biological environment. New advances in surface analysis instrumentation (e.g., new types of ion beams and mass analyzers for ToF-SIMS) hold the promise for extending our ability to characterize complex samples and extracting more detailed information about them.

### C. Characterization of nanoparticle surfaces

NPs are used in a variety of biomedical applications ranging from imaging to targeted delivery of therapeutics. NPs are attractive because of their high surface to volume ratio and their unique reactivities relative to large, bulk samples. However, until recently, surface characterization of NPs designed for biomedical applications has been lacking.^78–80^ Tools such as transmission electron microscopy (TEM), dynamic light scattering, localized surface plasmon resonance, etc. that can provide information about the shape, size distribution, and electronic properties have been widely used to characterize NPs,^81^ but they provide little or no information about the surface structure and composition of NPs. NPs are typically synthesized from multicomponent, complex mixtures that not only contain building blocks for the NPs (e.g., gold chloride for preparing AuNPs) but also a wide range of stabilization agents, surfactants, reactants, and contaminants that can be deposited onto the synthesized NPs.^56^ Unlike large, bulk samples that can easily be held by tweezers for rinsing and cleaning steps, purifying NPs can be challenging. The standard method of centrifugation and resuspension is basically a dilution process, which means that the original synthesis solution can be significantly diluted, but it is never completely separated from the NPs.^82^ Other methods such as dialysis can be used to achieve more complete removal of the species from the synthesis solution,^83^ but sometimes, dialysis will also remove some of the desirable species attached to the surface of the NPs.

**Fig. 7.**
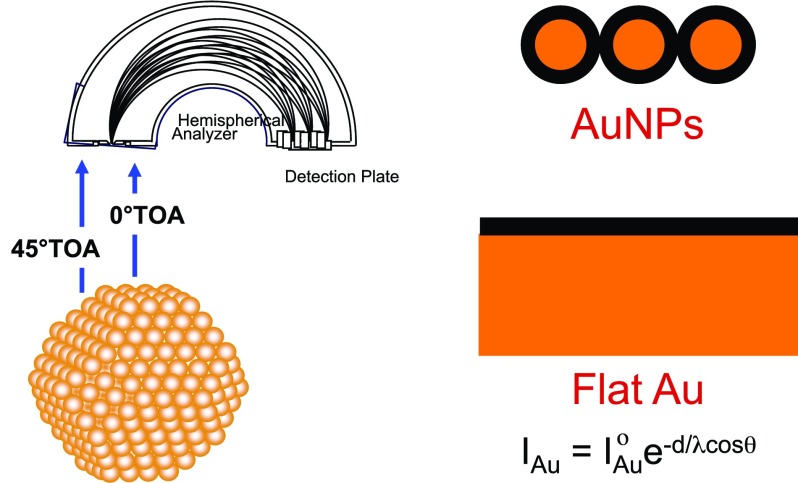
Schematic showing in XPS experiments on NPs the full range of photoelectron take-off angles detected, in comparison to the well-defined photoelectron take-off angle detected from flat surfaces.

Once the NPs are synthesized, the next step is typically surface functionalization. This usually involves the displacement of the coating deposited onto the NPs from the synthesis solution with another species that provides the desired surface properties for a particular application. For example, citrate covered AuNPs can be placed into a thiol solution to form a SAM on the AuNP surface. Often, the initial coating is not completely displaced.^84^ Mostly, the surface functionalization is a multistep process. For example, if the SAM covered AuNPs have a reactive terminal group on their surface, then biomolecules or other chemical species can be attached to the SAM covered AuNPs (e.g., thiolated DNA attached to a SAM with surface maleimide groups). Again, the extent of attachment as well as the deposition of unexpected species can be a problem. So, detailed surface analysis is needed in each step (synthesis, functionalization, biomolecule attachment, etc.) of the NP fabrication process. In spite of this significant need for surface analysis, most biomedical NP studies neglect the detailed surface analysis characterization of the NPs.^80^ Until recently, this was in part because the straightforward methodology for obtaining quantitative results from surface analysis of NPs was lacking. For example, XPS has been used for decades for accurately determining the overlayer thickness on flat surfaces.^85^ The equation used for this calculation relies on having a well-defined photoelectron take-off angle from the sample surface. In contrast, from spherical particles, the full range of photoelectron take-off angles (0°–90°) are detected (see Fig. 7), and so, different methodologies were required to analyze the XPS data. The catalysis field has a long history of addressing these challenges for XPS analysis of NPs. For example, a 1979 paper provides the methodology and equations needed to determine the average size of NPs on high surface area supports (>200 m^2^/g).^86^ The XPS results using this methodology have shown excellent agreement with NP sizes determined by complementary techniques such as TEM.^87–89^ Additional methods of quantifying XPS data were developed, including the methodology that could be extended to the analysis of NPs.^90^ However, these methodologies were computationally intensive, thus limiting their application to largely expert users. More recently, additional methods for analyzing XPS data from curved surfaces have been developed.^91–97^ Some still involve reasonable extensive calculations and simulations, while others are much less computationally intense. This has resulted in XPS being used to quantify overlayer thicknesses for SAM and protein covered NPs.^82,97–99^ Most of these samples are well-defined samples, such as nominally spherical AuNPs covered by well ordered SAMs (modeled as ideal spheres with concentric shell overlayers). However, recently, the methodology has been developed to incorporate deviations from ideality such as nonspherical shapes and offset cores.^100^ Thus, biomedical researchers now have good access to characterizing the composition and the thickness of overlayers on NPs. To date, XPS has been the main surface analysis technique used for NP characterization, with a focus on determining the overlayer composition and thicknesses.

Future challenges include extending the XPS analysis to more complex, multicomponent NP coatings as well as developing additional complementary surface analysis techniques such as ToF-SIMS for NP characterization, for example, extending the capabilities of ToF-SIMS developed for characterizing the structure of proteins immobilized onto a flat surface to proteins immobilized onto NP surfaces. The ultimate goal is to obtain a detailed surface characterization of functionalized NPs in solution, especially in biological environments.

### D. Imaging biological cells and tissue sections

**Fig. 8.**
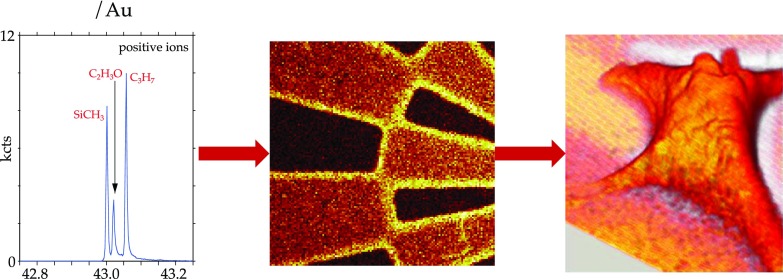
ToF-SIMS data in this figure show how over the past few decades biomedical surface analysis has evolved from spectroscopic analysis of homogeneous surfaces to 2D chemical state imaging of pattern surfaces to 3D imaging of biological cells.

**Fig. 9.**
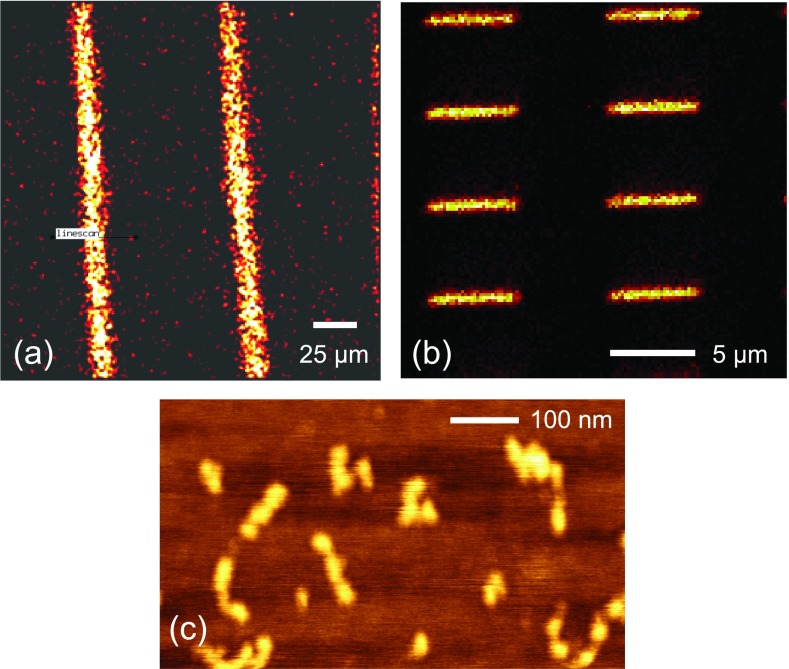
Examples showing different surface analysis techniques with different spatial resolution capabilities. (a) XPS Si_2p_ image of 5 *μ*m wide SiO_2_ lines separated by 80 *μ*m wide photoresist lines. (b) ToF-SIMS Au image of 0.24 × 5 *μ*m Au rectangles on a Si wafer. (c) AFM image of fibronectin molecules on a mica surface.

For biomedical devices, it is critical to determine the chemical state (composition, molecular structure, orientation, etc.) and distribution of biological and chemical moieties present on a surface as many of the important functions of cells and tissue depend on the arrangement of molecules at their surfaces.^101^ Thus, it is essential to develop surface analysis techniques capable of providing detailed chemical state information at high spatial and depth resolutions (i.e., chemical state 2D and 3D images). Using ToF-SIMS as an example, Fig. 8 summarises the development of surface analysis techniques from initially focusing on detailed spectroscopic analysis of homogeneous surfaces to then evolving into chemical state imaging of 2D patterned surfaces and more recently providing 3D images of biological materials. In addition to ToF-SIMS,^12,13,102–104^ there are a variety of other methods such as XPS,^105,106^ AFM,^107^ NEXAFS,^15^ SFG,^108^ and SPR (Ref. 16) that can acquire imaging data. Each of these techniques has its own strengths and weaknesses with respect to generating chemical state information and spatial/depth resolution. Together, they provide a powerful set of complementary techniques. For example as shown in Fig. 9, XPS has the lowest spatial resolution (∼5–10 *μ*m) but is the most quantitative and can be applied to virtually any material. Conversely, AFM has the highest spatial resolution (atomic to molecular) but typically provides limited chemical state or spectroscopic information. ToF-SIMS data contain the most detailed molecular structure information and have a spatial resolution that lies between XPS and AFM (0.1–1 *μ*m), but ToF-SIMS quantitation can be challenging. Thus, a complementary, multitechnique approach using XPS, ToF-SIMS, and AFM can investigate a biomedical device in a manner designed to combine the strengths of all the three techniques to obtain a more complete understanding of the device.

The central goal of modern bioengineering is the development of biomaterial surfaces that direct the biological healing response.^101^ These novel surfaces are envisioned to have a well-defined array of recognition sites designed to interact specifically with cells.^101^ The development of surface analysis techniques that will provide detailed chemical state information at a high spatial resolution is required for mapping out the presentation of these recognition sites on a biomaterial surface. In addition to mapping out the 2D location of chemical species on surfaces, there are many biomedical applications where imaging of 3D distributions of chemical and biological species is needed. Some examples include drug loading and release from polymers, biological species in tissue scaffolds, and nanoparticles in cells and tissue.

The commercial availability of reliable and stable C_60_ and gas cluster ion sources in the past ∼10 years has opened new possibilities for molecular depth profiling organic and biological materials.^109–111^ Model systems such as delta layers in organic films have been used to document the power of these sources to sputter through such films while introducing minimal residual damage, thus allowing molecular information to be obtained from profiles that can go microns into the sample.^112^ While both XPS and ToF-SIMS have been used in these studies, ToF-SIMS is the most widely used due to the detailed structural information that can be obtained from the molecular secondary ions sputtered from organic and biological materials. There have been two approaches to acquiring ToF-SIMS 3D images. One uses a dual primary ion beam approach with a cluster ion beam for sputtering and a liquid metal ion gun (LMIG) for analysis.^113^ The cluster ion beams remove materials, typically in 10–20 nm slices, while leaving little residual damage in the sample. The high mass resolution and high spatial resolution capabilities of LMIGs are then used to acquire 2D images after each sputter cycle. However, LMIGs do produce residual damage in organic and biological samples, and so, the ion dose of the LMIG analysis cycle must be limited to only a few percent of the ion dose of the sputter cycle to allow the sputter cycle to remove the damage produced by the analysis beam.^113^ To obtain high quality images from biological cells so that subcellular features can be identified and visualized, both good spatial resolution (∼1 *μ*m or better) and good mass resolution (several thousand m/Δm) are required.^114^ Historically in pulsed LMIGs, there was a trade-off in mass resolution for spatial resolution (e.g., pulsing the LMIG improved the mass resolution but degraded the spatial resolution). Recently, operational modes of pulsed LMIGs have been optimized to provide images with both high mass and spatial resolutions, but this can result in longer image acquisition times. Thus, a ToF-SIMS instrument that used a direct current primary ion beam and pulsed the secondary ions was developed.^115^ This allowed images and spectra with both high mass and spatial resolutions to be rapidly acquired. It also allowed a wider range of primary ion beams, including cluster ion beams, to be used for the acquisition of the high mass and spatial resolution images and spectra. The addition of ms/ms capability to ToF-SIMS instruments in recent years has added even more power to the technique for 3D imaging of biological materials.^115,116^

The ongoing advances being made in the capabilities of ToF-SIMS for 2D and 3D imaging is currently being used by researchers to push the limits of what information can be obtained from biological materials. For example, ToF-SIMS can distinguish between different types of cells.^117,118^ It can also be used to identify different regions (tumor, stroma, necrotic, etc.) of breast tissue sections as well as the distribution of key lipids and fatty acids.^119–121^ These results provide insight into the metabolic state of cells in the tissue sections and the effectiveness of various regimes used to treat the cancer tumors. To date, most of these studies have combined ToF-SIMS imaging with histology images. Other studies have also included other imaging modalities such as matrix assisted laser desorption/ionization (MALDI).^122,123^ Just as in spectroscopic biomedical surface analysis where it is essential to use a complementary, multitechnique approach to obtain a detailed understanding of the surface structure and composition, it is also important to apply a multimodal approach to imaging cells and tissue sections. Thus, it is anticipated that approaches that combine imaging modalities such as histology, ToF-SIMS, MALDI, atmospheric mass spectrometry, and confocal microscopy will become more common in the future. Each of these methods has different strengths and weaknesses. Thus, in combination, they will provide a more comprehensive analysis of biological cells and tissue sections.

Although the developments and advances in ToF-SIMS have been continuous and impressive over the past 20 years, there are still challenges that need to be addressed to fully exploit this technique for 2D and 3D analysis of biological materials. Experimental procedures (sample preparation and data acquisition) require further optimization.^124,125^ One example is developing methods to prepare tissue sections for analyses that leave all biological species such as lipids and cholesterol in the same state and location as they were the native, live tissue. Also, the ToF-SIMS signals from tissue sections can change significantly with time, and so, another challenge is to find a sample preparation method that stabilizes the samples, allowing the samples to be shipped between laboratories for analysis. Finally, 3D imaging of tissue sections is still challenging due to secondary ion suppression effects as the sample is sputtered. There are still challenges for efficient processing of the large amounts of data acquired in 3D imaging.^22,126^ Examples include correcting for the different sputter rates of different species in multicomponent samples^127^ and easily extracting and visualizing all the relevant information present in these large datasets.^22^ Due to limitations in the secondary ion yields, the ToF-SIMS community is always working on developing new primary ion sources, more efficient mass analyzers, etc., to address this situation.^104,128^ Today's state-of-the-art ToF-SIMS instruments provide impressive capabilities in terms of sample handling, primary ion beams, mass analyzers, and ms/ms options, each with their own advantages and disadvantages in terms of the secondary ion yield, spatial resolution, mass resolution, image acquisition time, etc. These capabilities should lead to further advances in our ability to acquire 2D and 3D chemical state images.

## III. SUMMARY

Significant advances in biomedical surface analysis have been realized over the past ∼35 years. This has come from a combination of developing new instrumentation, experimental protocols, and data analysis methods. Both advances in a given technique such as SIMS and development of complementary, multitechnique analysis have resulted in a powerful set of tools that provide comprehensive surface characterization from a wide range of biomaterials used in biomedical applications. Although an impressive level of detail can now be obtained about the structure and composition of surfaces and interfaces, as well as biomolecules interacting with those surfaces and interfaces, there are still many challenges for biomedical surface analysis to address. There is still no atomic level structure of a surface bound protein in the Protein Data Bank. There is still a need to develop new methods for obtaining detailed surface structure of composition of NPs in solution. Many of the advances made have used model surfaces and systems to develop the surface analysis methodology. These advances must continue and be applied to increasingly complex, multicomponent biomedical devices. The recent successes in 3D imaging are particularly exciting, but still require significant development to address the challenges that still exist (sample preparation, differential sputter rates, imaging processing, etc.) as well as fully implementing a multimodal imaging approach.
